# First record of mermithid parasitism in adult biting midges, *Culicoides huffi* (Diptera: Ceratopogonidae), collected from Southern Thailand, with ultrastructural and molecular characterization

**DOI:** 10.1186/s13071-025-06958-x

**Published:** 2025-07-28

**Authors:** Chulaluk Promrangsee, Vivornpun Sanprasert, Arunrat Thepparat, Sakone Sunantaraporn, Rinnara Ampol, Rungfar Boonserm, Padet Siriyasatien, Kanok Preativatanyou

**Affiliations:** 1 Interdisciplinary Program of Biomedical Sciences, Graduate School, Chulalongkorn University, Bangkok, Thailand; 2 Department of Parasitology, Faculty of Medicine, Chulalongkorn University, Bangkok, Thailand; 3 Department of Agricultural Technology, Faculty of Science, Ramkhamhaeng University, Bangkok, Thailand; 4 Center of Excellence in Vector Biology and Vector-Borne Disease, Chulalongkorn University, Bangkok, Thailand

**Keywords:** Mermithid parasitism, *Culicoides huffi*, Biting midges, Intersexuality, Biological control, Southern Thailand

## Abstract

**Background:**

*Culicoides* biting midges are known vectors of several pathogens, including arboviruses, protozoa, and filarial worms. Additionally, mermithid nematodes have been found to parasitize *Culicoides* midges, causing pathogenic effects that reduce host fitness and often lead to death. Consequently, mermithids have potential as biocontrol agents to reduce midge populations. However, the biology of these entomopathogenic nematodes infecting *Culicoides* in Thailand remains unknown.

**Methods:**

As part of the leishmaniasis surveillance program in Nakhon Si Thammarat Province, Southern Thailand, we collected *Culicoides* midges near the residence of a leishmaniasis patient in November 2024. The *Culicoides* samples were morphologically identified and examined microscopically for parasitic nematodes. Infected midges were dissected to isolate nematodes from each individual. The nematodes were then characterized morphologically using scanning electron microscopy (SEM) and identified molecularly via polymerase chain reaction (PCR) targeting the non-filarial hypervariable region I of the small subunit ribosomal RNA gene (SSU HVR-I) and mermithid small subunit ribosomal RNA (SSU rRNA) gene, followed by nanopore sequencing, phylogenetic analysis, and species delimitation testing.

**Results:**

A total of 263 field-caught adult *Culicoides* midges were collected, with *Culicoides huffi* of the *Calvipalpis* group being the most abundant species (*n* = 155, 58.9%). Among these, 35 *C. huffi* samples, including 4 males, 11 females, and 20 intersex males, were infected with nematodes, resulting in an overall infection rate of 13.3%. The parasitized intersex males, presumably genetically male, exhibited a high degree of feminization in their antennae and wings, which likely enhances female-like behaviors such as detecting and flying toward breeding sites, possibly facilitating parasite transmission and reproductive success. The SEM analysis revealed key morphological features consistent with parasitic nematode larvae of the Mermithidae family, including a long, slender body, a stylet, cephalic papillae, amphids, and a trophosome. Basic Local Alignment Search Tool for nucleotide (BLASTn) analysis of non-filarial SSU HVR-I and mermithid-specific SSU rRNA gene sequences identified all nematodes as mermithids, showing 94.2–94.4% similarity to *Pheromermis* sp. from the hornet and 97.3% similarity to *Mermis* sp. from *Culicoides obsoletus*. Phylogenetic analysis and species delimitation suggest that these sequences represent a single putative species distinct from other known mermithids.

**Conclusions:**

This study is the first to report mermithid parasitism in *Culicoides* midges in Thailand, incorporating both ultrastructural and molecular characterization. The novel morphological and molecular insights provided here contribute to a more comprehensive understanding of the biology of entomopathogenic nematodes. Further research is needed to evaluate the potential of these nematodes for the biological control of *Culicoides* biting midges.

**Graphical Abstract:**

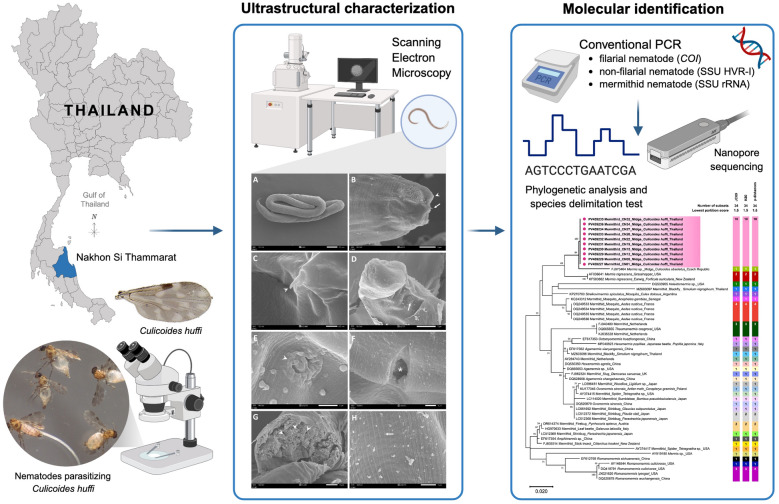

**Supplementary Information:**

The online version contains supplementary material available at 10.1186/s13071-025-06958-x.

## Background

*Culicoides* biting midges are hematophagous insects belonging to the family Ceratopogonidae, order Diptera, with more than 1360 known species recorded worldwide [1, 2]. These insects are involved in the transmission of several diseases affecting domestic and wild animals, as well as humans [3]. They transmit a variety of pathogens, including arboviruses, protozoa, and filarial worms. Important arboviruses transmitted by *Culicoides* biting midges include African horse sickness virus in equids; bluetongue, epizootic hemorrhagic disease, and Schmallenberg viruses in ruminants; Oropouche virus in humans; and vesicular stomatitis virus, primarily affecting horses and cattle [3–5]. Protozoan parasites transmitted to birds by *Culicoides* include *Haemoproteus*, *Leucocytozoon*, and *Trypanosoma* [6–8]. Filarial nematodes of the genera *Dipetalonema*, *Onchocerca*, and *Mansonella* can parasitize *Culicoides* midges, allowing the nematodes to develop to an infective stage and be transmitted to vertebrate hosts, including birds, livestock, wildlife, and humans [4, 9].

More than 168 *Culicoides* species have been documented in Southeast Asia, with approximately 100 described in Thailand [10]. Notably, several livestock-associated *Culicoides* species in Thailand are known potential vectors of bluetongue virus, including *Culicoides peregrinus*, *Culicoides orientalis*, *Culicoides imicola*, *Culicoides oxystoma*, and *Culicoides fulvus* [11]. Meanwhile, *Culicoides mahasarakhamense* and *Culicoides guttifer* are vectors of avian leucocytozoonosis [12]. Interestingly, DNA of two *Leishmania* (*Mundinia*) species, namely *Leishmania martiniquensis* and *Leishmania orientalis*, has been detected in several *Culicoides* species collected from leishmaniasis-endemic areas of Thailand. This suggests that these species could be potential vectors of the disease in the country [13–17]. This speculation was supported by previous experimental evidence showing that these *Leishmania* parasites can infect and develop into infective metacyclic promastigotes in laboratory-infected *Culicoides sonorensis* [18–20].

In addition to filarial nematodes, mermithids, another group of nematodes belonging to the Mermithidae family, can parasitize biting midges. Several species of biting midges from different geographical regions have been reported to be infected by mermithids [21]. Beyond biting midges, mermithids also infect other dipteran insects such as mosquitoes [22] and black flies [23, 24], as well as various insects from the orders Lepidoptera [25], Orthoptera [26], Coleoptera [27], Hemiptera [28], and Hymenoptera [29]. Due to their ability to sterilize or otherwise kill insect larvae or adults, mermithids have been utilized as biocontrol agents [30–32]. Infection by mermithids can induce significant morphological changes, including feminization of males and the loss of functional male reproductive organs, resulting in infertility [33, 34]. Additionally, behavioral changes and increased physiological stress may occur, leading to reduced host fitness and higher mortality [35, 36].

In recent years, we have investigated the role of *Culicoides* midges in the transmission cycle of leishmaniasis through microscopic dissection and molecular detection of *Leishmania* parasites in midge samples collected from various endemic locations in Northern and Southern Thailand. Notably, some midge samples collected from a leishmaniasis focus in Nakhon Si Thammarat Province, Southern Thailand were parasitized by worms whose external morphology matched that of mermithid nematodes. This finding highlights the need for further morphological and molecular studies of these entomopathogenic nematodes.

In the present work, we characterized mermithid nematodes isolated from *Culicoides* samples collected from Nakhon Si Thammarat Province using scanning electron microscopy (SEM) and sequence analysis of the small subunit ribosomal RNA (SSU rRNA) gene. We also analyzed the phylogenetic relationships and species delimitation of our mermithid sequences in comparison with other known mermithid species. The novel ultrastructural and molecular data obtained from this study will improve our understanding of the biology of midge-associated mermithid nematodes, a group that remains largely understudied.

## Methods

### Investigation area, *Culicoides* midge collection, and morphological species identification

**Fig. 1:**
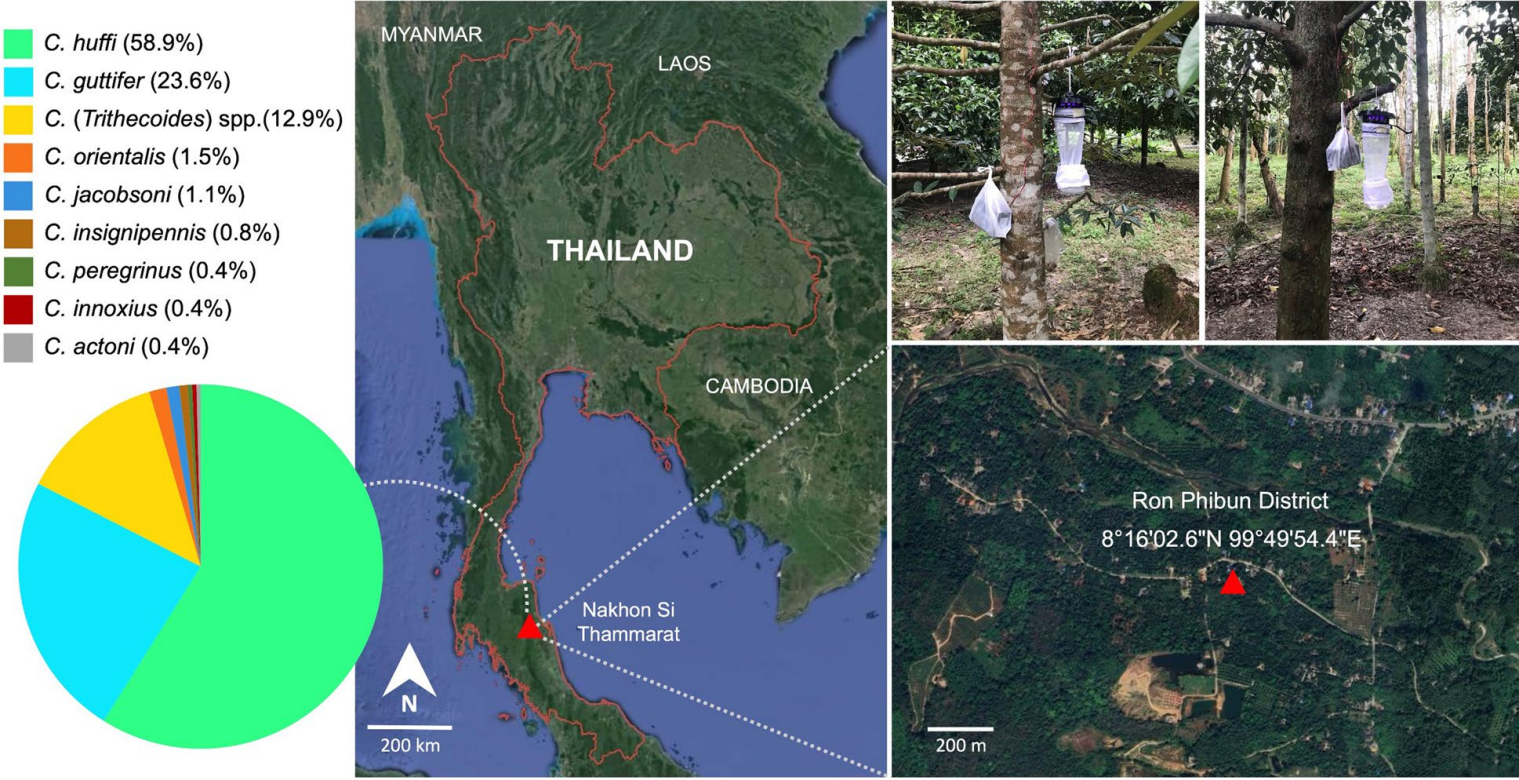
Geographical location of the midge sampling site in Ron Phibun District, Nakhon Si Thammarat Province, Southern Thailand. Light traps were installed in the residential area of a patient with cutaneous leishmaniasis. The colored pie chart shows the relative abundance of each *Culicoides* species collected. The map and magnified satellite image were adapted from public domain sources (https://maps.google.com and https://earth.google.com)

*Culicoides* biting midges were collected in November 2024 from a durian plantation adjacent to the residential area of a patient recently diagnosed with localized cutaneous leishmaniasis caused by *Leishmania orientalis* in Ron Phibun District (8°16′02.6″ N, 99°49′54.4″ E), Nakhon Si Thammarat Province, Southern Thailand, as depicted in Fig. 1. The Mahidol University ultraviolet light-emitting diodes (MU UV-LED) light traps were placed approximately 1 m above the ground and operated from 6 p.m. to 6 a.m. for two consecutive nights. The following day, the captured insects were anesthetized in a freezer for 20 min to facilitate specimen handling. Midges were separated from the insect collection on the basis of characteristic wing venation and pigmentation [10]. The sexes of *Culicoides* individuals were determined by distinct morphological features, including antennal patterns, the presence of spermathecae, and male genitalia. All midges were then morphologically identified under a stereomicroscope (EZ4 HD, Leica, Germany) on the basis of wing pigmentation patterns, following the identification keys for *Culicoides* species in Southeast Asia [10] and the morphological description of the novel species *C. mahasarakhamense* [37].

### Dissection of *Culicoides* midges and measurement of nematode parasites

*Culicoides* individuals were examined under a stereomicroscope (EZ4 HD, Leica, Germany) for the presence of parasitic nematodes. When a nematode was observed in the abdominal cavity of a midge, the abdominal wall was carefully dissected under the microscope to remove the worm. Dissection was performed in a drop of phosphate-buffered saline (pH 7.4) on a sterile glass slide under a stereomicroscope. The recovered nematodes were then visualized and photographed at 100× magnification with an Olympus CX43 light microscope equipped with an Olympus SC180 digital camera (Olympus, Tokyo, Japan). The body size (total length and mid-body width) of the nematodes was measured using OLYMPUS cellSens imaging software version 4.3.1 (Olympus, Tokyo, Japan) and ImageJ software [38].

### Ultrastructural characterization of nematodes by scanning electron microscopy

After measurement, the nematodes were prepared for SEM by fixation in a 2.5% glutaraldehyde solution in 0.1 M phosphate buffer (pH 7.4) for 1 day, followed by dehydration through a graded ethanol series. The dehydrated samples were then dried using a critical point dryer and mounted on stubs with double-sided adhesive tape prior to gold coating. The samples were gold-coated using a Balzers SCD 040 sputter coater (Oerlikon Balzers, Germany) and subsequently examined with a JSM-IT500HR scanning electron microscope (JEOL, Tokyo, Japan) at an accelerating voltage of 10 kV, with images captured accordingly.

### DNA extraction and whole genome amplification of nematodes

After SEM analysis, the nematodes attached to the double-sided adhesive tapes were individually transferred into separate microcentrifuge tubes containing 100 µL of acetone solution to detach the worms from the tape. Once completely dried, 200 µL of lysis buffer containing Proteinase K was added to each nematode sample, which was then incubated overnight at 50 °C. The lysate was subsequently purified using the DNeasy Blood & Tissue Kit (Qiagen, Hilden, Germany) following the manufacturer’s protocol. The extracted DNA samples underwent whole genome amplification (WGA) of genomic DNA (gDNA) using the REPLI-g kit (Qiagen, Hilden, Germany), which increased the quantity and concentration of nematode DNA with high fidelity. The concentration of the amplified DNA samples was assessed using the Qubit^™^ dsDNA High Sensitivity Assay Kit (Thermo Fisher Scientific, MA, USA) on a Qubit^™^ 4 fluorometer.

### Conventional PCRs for nematode species identification

Molecular identification of the unknown nematode samples was performed using polymerase chain reactions (PCRs) with primers previously designed to detect the filarial cytochrome c oxidase subunit I gene (*COI*), the non-filarial hypervariable region I of the SSU rRNA gene (SSU HVR-I), and the mermithid SSU rRNA gene, followed by nanopore sequencing and Basic Local Alignment Search Tool for nucleotide (BLASTn) analysis. Information on the oligonucleotide primers and expected amplicon size is provided in Table 1. Briefly, PCR reactions were carried out in a 50 μL mixture containing 25 μL of 2× KAPA HiFi HotStart ReadyMix (Roche, Basel, Switzerland), 2 μL of each 10 μM primer, 5 μL of WGA-enriched gDNA, and 16 μL of nuclease-free water. The amplification conditions consisted of an initial denaturation at 95 °C for 5 min, followed by 40 cycles of 98 °C for 30 s; annealing at 48 °C for 30 s (filarial *COI*), 50 °C for 30 s (non-filarial SSU HVR-I), or 50 °C for 30 s (mermithid SSU rRNA); extension at 72 °C for 1 min; and a final extension at 72 °C for 5 min. PCR amplicons were verified by 1.5% (*w*/*v*) agarose gel electrophoresis using the GENESTA^™^ 100 bp DNA ladder marker (GeneAll^®^, Seoul, South Korea), stained with ethidium bromide (Promega Corporation, WI, USA), and visualized using the GelDoc Go Imaging System (Bio-Rad, CA, USA). All PCR products were subsequently purified individually using Agencourt AMPure XP beads (Beckman Coulter, CA, USA).

**Table 1:** Oligonucleotide primers used for molecular identification of nematodes in this study

Species/gene target	Primer name	Oligonucleotide sequence (5′ → 3′)	Amplicon size (bp)	Reference
Filarial nematode				
* COI*	COIintF	TGATTGGTGGTTTTGGTAA	689	[39]
	COIintR	ATAAGTACGAGTATCAATATC		
Non-filarial nematode				
SSU HVR-I	RH5401	AAAGATTAAGCCATGCATG	919	[40]
	RH5402	CATTCTTGGCAAATGCTTTCG		
Mermithid nematode				
SSU rRNA gene	Merm forward	CAAGGACGAAAGTTAGAGGTTC	802	[41]
	Merm reverse	GGAAACCTTGTTACGACTTTTA		

### MinION^®^ sequencing and molecular identification of nematodes

Purified amplicons of each target were quantified using the Qubit^™^ dsDNA High Sensitivity Assay Kit (Thermo Fisher Scientific, MA, USA). The amplicons were then end-repaired using NEBNext^®^ Ultra^™^ II End Repair/dA-Tailing Module (New England Biolabs, MA, USA) and ligated with native barcodes from the Native Barcoding Kit 96 V14 (Cat. No. SQK-NBD114.96, Oxford Nanopore Technologies, Oxford, UK) using the NEB Blunt/TA Ligase Master Mix (New England Biolabs, MA, USA). All barcoded amplicon samples were pooled and subjected to sequencing adaptor ligation using the NEBNext Quick Ligation Module (New England Biolabs, MA, USA). After purification with AMPure magnetic beads, 50 fmol of the purified library was loaded onto a MinION^®^ R10.4.1 flow cell and sequenced overnight. Base calling was performed using the super-accuracy model in Dorado version 7.3.11. Low-quality reads with *Q* scores below 20 were filtered out using Chopper version 0.7.0 [42]. Consensus sequence analysis was conducted with amplicon_sorter.py (version 2024_02-20) [43]. The resulting consensus sequences were aligned against reference sequences in GenBank using the Basic Local Alignment Search Tool for nucleotide (BLASTn) [44] for taxonomic assignments. The nucleotide sequences obtained were submitted to the GenBank database. Nematode sequences from previous consensus analyses, along with reference sequences from GenBank, were aligned using ClustalW implemented in BioEdit version 7.2.6 [45] prior to phylogenetic analysis.

### Phylogenetic analysis and species delimitation test

The sequence alignment of non-filarial SSU HVR-I and mermithid SSU rRNA gene sequences obtained in this study, together with their respective reference sequences from GenBank, including mermithid SSU HVR-I and mermithid SSU rRNA gene sequences previously identified in other insect species (Supplementary Files 1 and 2), was phylogenetically analyzed using MEGA11 software [46]. The maximum likelihood method was applied with the Kimura two-parameter (K2P) model incorporating gamma distribution and invariant sites (K2P + G + I) for the non-filarial SSU HVR-I locus, and the Kimura two-parameter model with discrete gamma distribution (K2P + G) for the mermithid SSU rRNA gene, as these were identified as the best substitution models. Bootstrap analysis was performed with 1000 replicates to assess branch support.

Species delimitation tests were conducted on the basis of both the non-filarial SSU HVR-I and mermithid SSU rRNA gene sequences using Assemble Species by Automatic Partitioning (ASAP) [47]. Three substitution models were applied: Jukes-Canter (JC69), Kimura (K80), and simple distance (*p*-distances). The partitioning with the lowest partition score was considered the best-performing delimitation result.

## Results

### Collection and identification of *Culicoides* midges with microscopic screening of nematodes

**Fig. 2:**
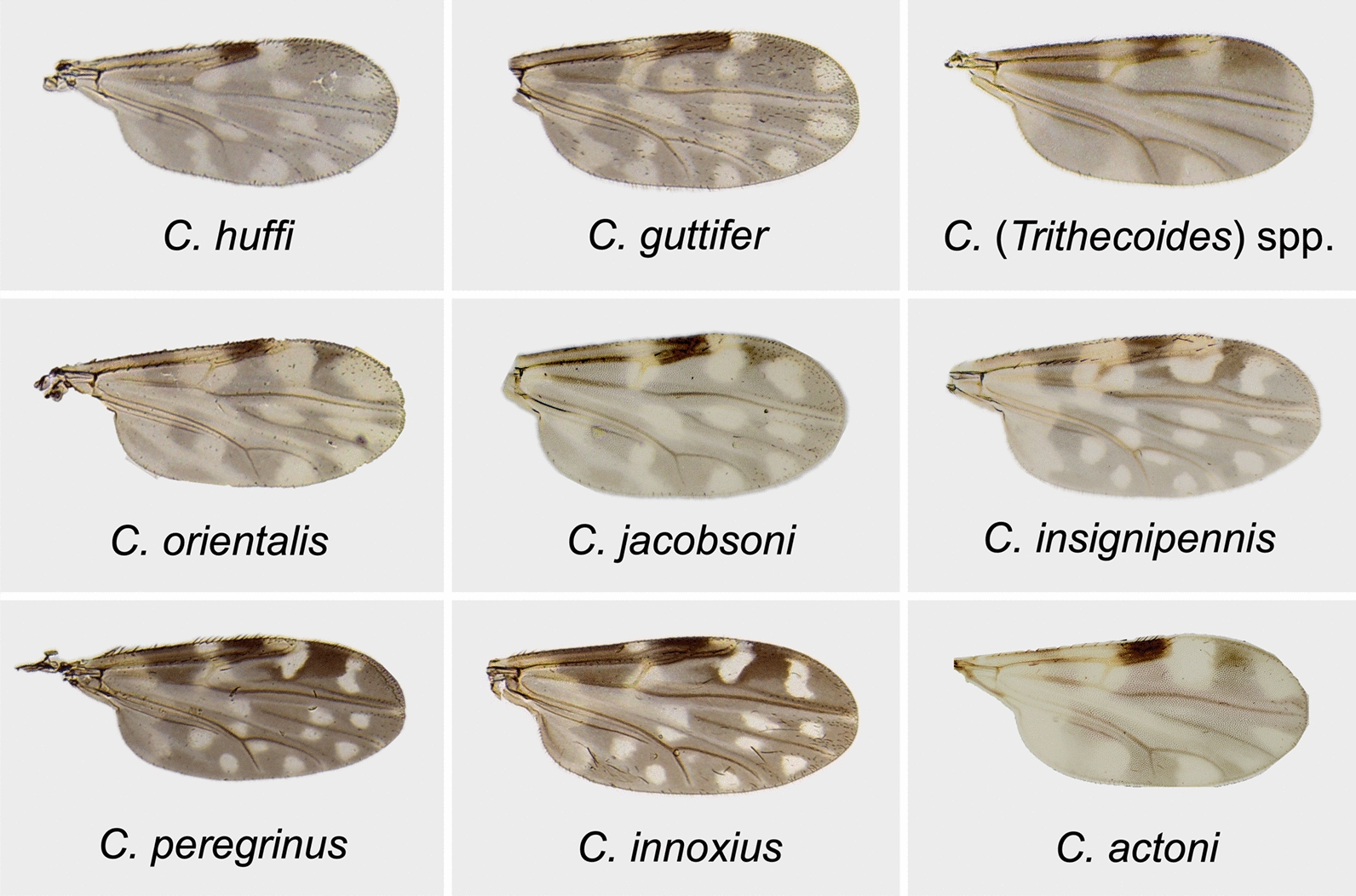
Wing pigmentation patterns of *Culicoides* midge species collected from the study area, ranked from highest to the lowest relative abundance

A total of 263 *Culicoides* specimens were collected. Using the wing pigmentation patterns as key morphological characters, the *Culicoides* specimens were identified as belonging to at least nine species, with individuals of different sexes distinguished (Fig. 2). The two most abundant species were *C. huffi* (*n* = 155, 58.9%), followed by *C. guttifer* (*n* = 62, 23.6%). The majority of specimens were female (*n* = 234). Male specimens were recorded for only two species: *C. huffi* (*n* = 8) and *C. guttifer* (*n* = 1). Notably, 20 intersex males of *C. huffi*, characterized by male genitalia but female-like antennae, were also recorded. No intersex individuals with female genitalia were found.

**Fig. 3:**
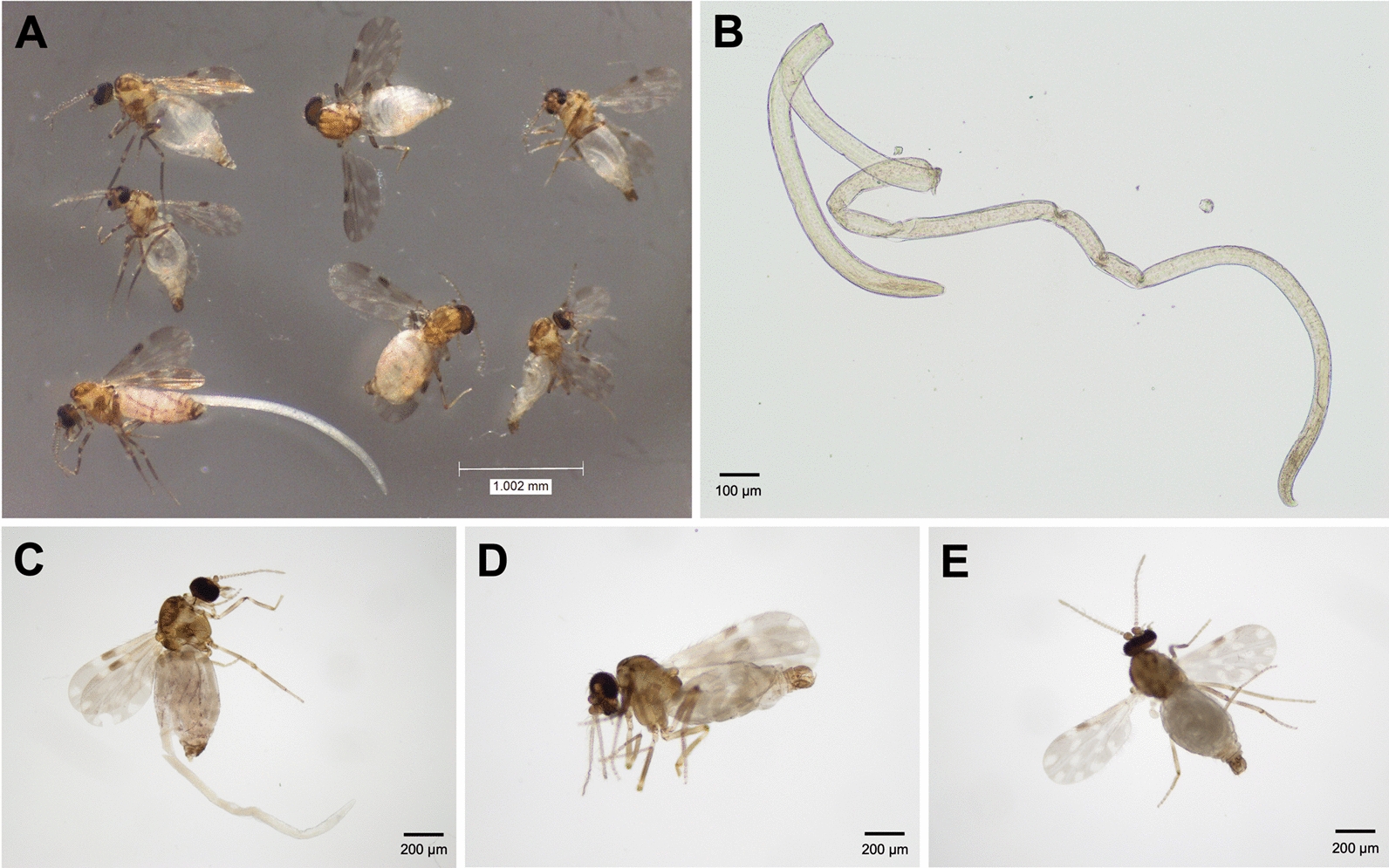
*Culicoides huffi* specimens infected with nematode parasites: **A** infected midges; **B** a mermithid nematode isolated from *C. huffi*. Photomicrographs of parasitized *C. huffi* of different sexes, including **C** a female with a post-parasitic juvenile emerging from the abdomen, **D** a male, and **E** an intersex male exhibiting male genitalia but female-type antennae

Nematodes were detected in 35 *C. huffi* specimens through abdominal dissection or emergence from the abdomen, including 4 males, 11 females, and all 20 intersex males, as shown in Fig. 3. The overall prevalence of nematode infection was 13.3% (35/263). Among the infected midges, three females and one intersex *C. huffi* individual harbored two worms each, whereas the remainder contained only one, totaling 39 nematodes.

**Fig. 4:**
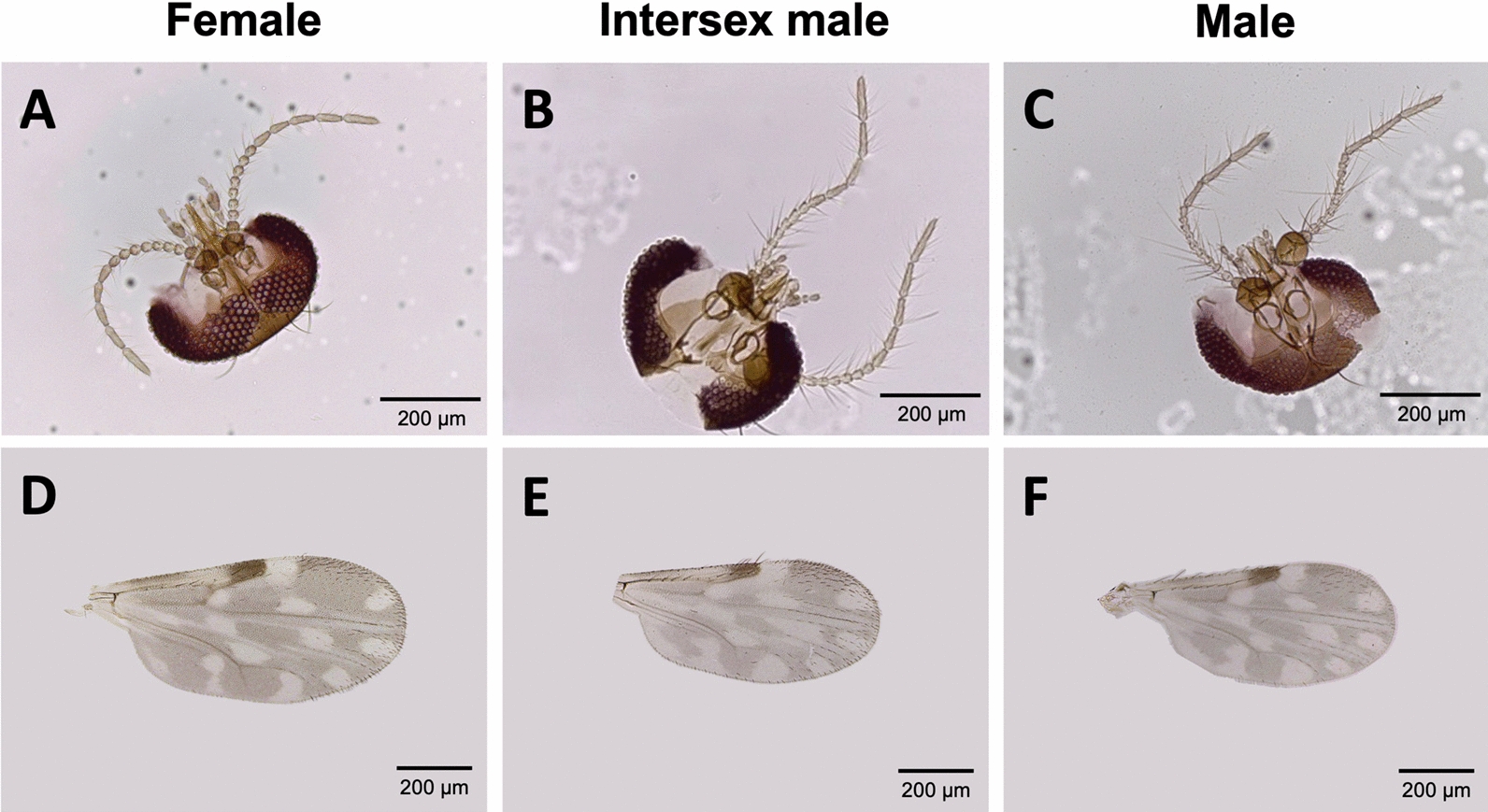
Antennae of female (**A**), intersex male (**B**), and male (**C**) *Culicoides huffi* infected with mermithid nematodes in this study. Wing size and shape of female (**D**), intersex male (**E**), and male (**F**) *C. huffi*. A high degree of feminization of the antennae and wing shape was observed in intersex individuals. All scale bars represent 200 µm in both antenna and wing micrographs

**Fig. 5:**
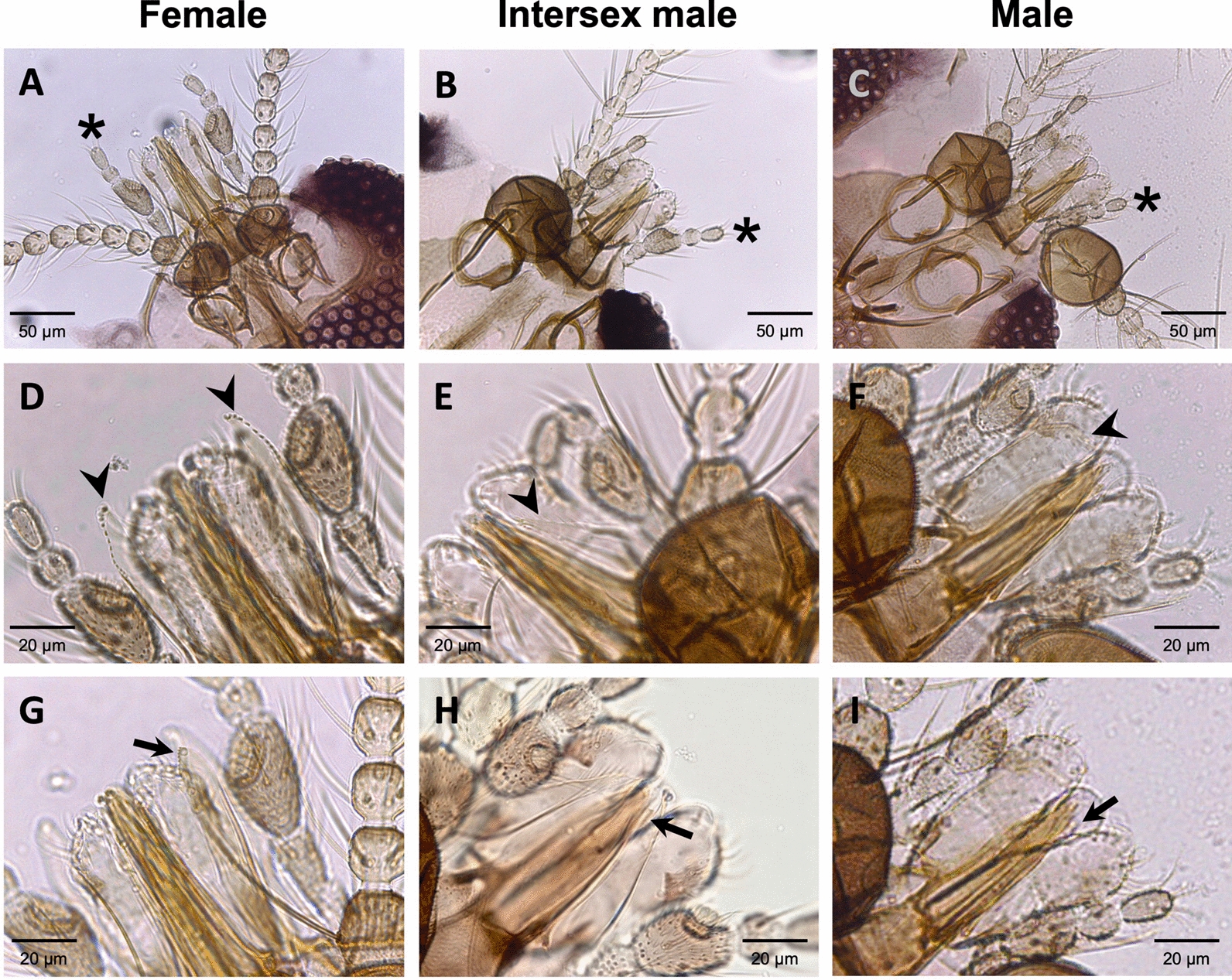
Micrographs of mouthparts from female (**A**, **D**, **G**), intersex male (**B**, **E**, **H**), and male (**C**, **F**, **I**) mermithid-infected *Culicoides huffi* in this study. Maxillary palps, laciniae, and mandibles are indicated by asterisks, arrowheads, and arrows, respectively. Unlike the antennae and wing shape, the mouthparts of intersex individuals exhibit intermediate morphology with varying degrees of feminization. All scale bars represent 20 µm

It was observed that the size and shape of the antennal segments of parasitized intersex males closely resembled those of females, featuring five distal elongate and eight proximal subspherical flagellomeres (Fig. 4A–C). Although female *C. huffi* had wings that were wider and differently shaped compared with males, intersex males exhibited wing sizes similar to males, but with wing shapes nearly identical to females (Fig. 4D–F). Additionally, the mouthparts of parasitized intersex males showed intermediate or partial feminization. The maxillary palp segments of intersex males were more similar to females, particularly the third palpal segment, which displayed intermediate swelling and a well-developed sensory pit filled with numerous sensilla, unlike the male pattern (Fig. 5A–C). The mandibles of intersex individuals were also modified toward the female form, unlike the less developed male mandibles. However, they possessed only 5–6 tiny teeth, compared with the 9–11 cutting teeth found in females (Fig. 5G–I). The laciniae of intersex forms differed in tooth number and shape, bearing 9–10 widely spaced, slender spicules, in contrast to females, which had 11–12 closely packed, well-developed teeth (Fig. 5D–F). The external genitalia of intersex samples resembled those of males. Morphological changes in the remaining parasitized males and females were unremarkable. Information on the species richness, sex distribution, and relative abundance of the collected *Culicoides* midges is provided in Table 2.

**Table 2:** Species composition and relative abundance of *Culicoides* biting midges collected in this study

Genus (subgenus/species group)	Species	Number of *Culicoides* without nematodes		Number of *Culicoides* parasitized with nematodes			Total
		Male	Female	Male	Female	Intersex	
*C.* (*Calvipalpis* group)	*huffi*	4	116	4	11	20	155 (58.9%)
*C.* (*Meijerehelea*)	*guttifer*	1	61	—	—	—	62 (23.6%)
*C.* (*Trithecoides*)	spp.	—	34	—	—	—	34 (12.9%)
*C.* (*Avaritia*)	*orientalis*	—	4	—	—	—	4 (1.5%)
*C.* (*Avaritia*)	*jacobsoni*	—	3	—	—	—	3 (1.1%)
*C.* (*Hoffmania*)	*insignipennis*	—	2	—	—	—	2 (0.8%)
*C.* (*Hoffmania*)	*peregrinus*	—	1	—	—	—	1 (0.4%)
*C.* (*Hoffmania*)	*innoxius*	—	1	—	—	—	1 (0.4%)
*C.* (*Avaritia*)	*actoni*	—	1	—	—	—	1 (0.4%)
Total		5	223	4	11	20	263

### Microscopic examination of parasitic nematodes and scanning electron microscopy

**Fig. 6:**
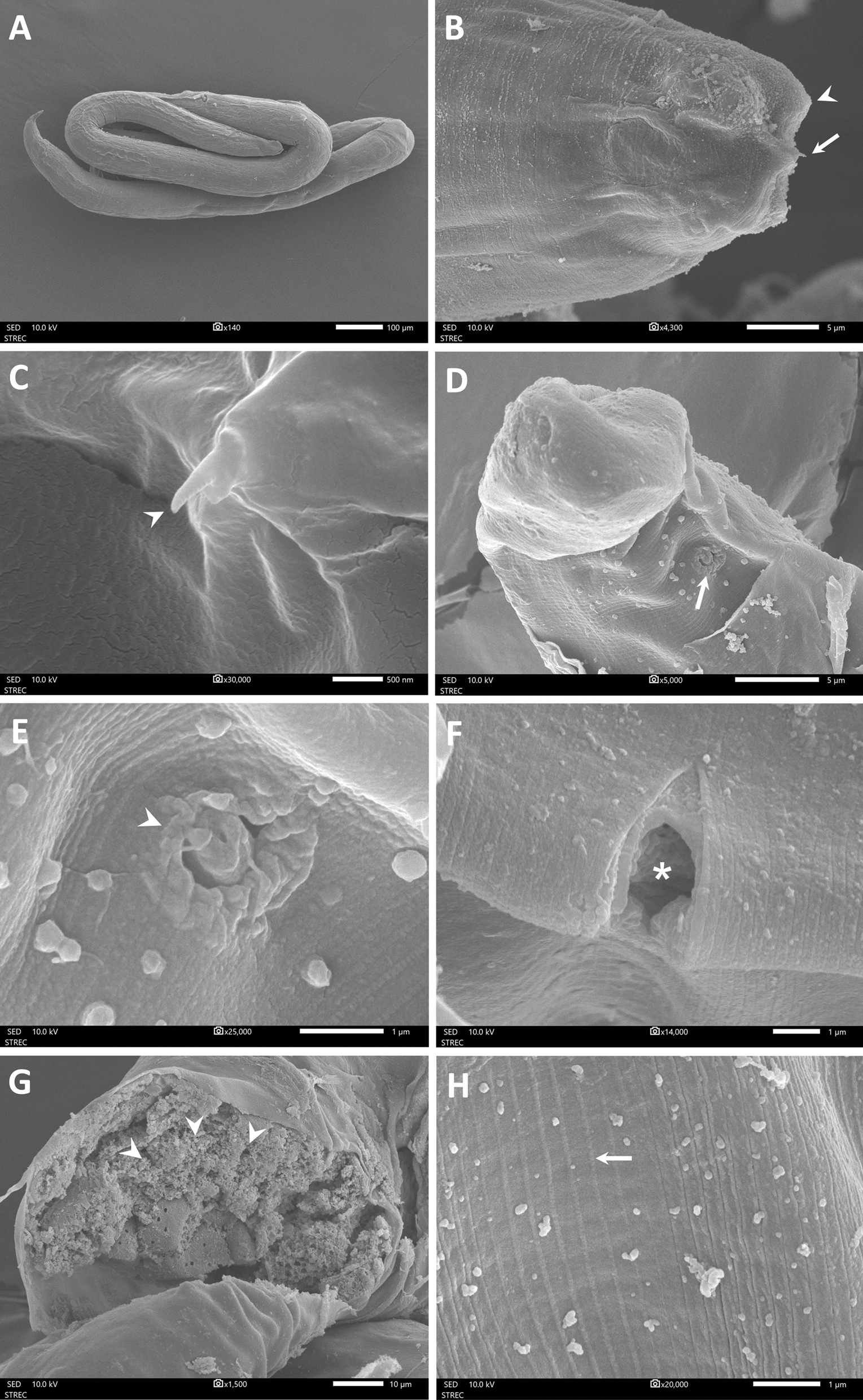
SEM photographs of mermithid nematodes: **A** Whole coiled worm. **B** Anterior part of the worm showing the cephalic papillae (arrowhead) and the stylet (arrow) at the anterior tip. **C** Enlarged view of B highlighting the stylet (arrowhead). **D** Amphid (arrow) on the lateral side of the pharyngeal region. **E** Enlarged view of D showing the amphidial aperture. **F** Lateral view of the vulva region (asterisk). **G** Transverse view of the worm body showing the trophosome containing lipoprotein globules (arrowhead). **H** Cuticle with superficial transverse annulations (arrow)

Of 39 nematode individuals, 18 were successfully recovered intact from the insect necropsies and measured, with a mean total length of 2901.2 µm and a mean body width of 38.4 µm. Only 12 individuals were successfully processed for SEM analysis. Examination of the SEM images revealed external morphological features that were not visible under light microscopy. The nematodes exhibited an elongated, slender body with a narrow, pointed head and a smooth cuticle (Fig. 6A). At the anterior end, cephalic papillae and a needle-like stylet were visible, indicating the juvenile stage, with no distinct mouthpart structures observed (Fig. 6B and C). Small chemosensory structures, or amphids, appeared as lateral annular depressions approximately 1 µm in diameter, featuring radial cuticular folds near the anterior end (Fig. 6D and E). The posterior end displayed a transverse slit-like vulva with a slightly ridged cuticular margin, located ventrally at the mid-body of the worm (Fig. 6F). Transverse sections revealed internal structures consisting of a trophosome with numerous storage globules (Fig. 6G). Superficial cuticular annulations were observed along the body surface (Fig. 6H). Overall, the morphological characteristics observed in the SEM images are consistent with third-stage juveniles (J3) that develop within the host’s body cavity, supporting the identification of the nematodes as developing parasites of the Mermithidae family.

### Molecular identification, phylogenetic analysis, and species delimitation of nematodes

Of 12 WGA-enriched gDNA samples, 10 were successfully amplified using two primer pairs targeting the non-filarial SSU HVR-I and the mermithid SSU rRNA gene. The filarial *COI*-PCR yielded negative results in this study, confirming that the samples did not belong to filarial taxa, which are typically found in the head and thorax of midges [48]. After nanopore sequencing and filtering of low-quality reads, a total of 153,052 and 84,395 high-quality reads were obtained for the non-filarial SSU HVR-I and mermithid SSU rRNA gene loci, respectively, from all amplified samples. These high-quality reads were analyzed to generate consensus sequences. On the basis of BLASTn results, all ten SSU HVR-I sequences exhibited 94.2–94.4% similarity to the *Pheromermis* sp. reference (KR029620) [49], previously identified in the hornet (*Vespa velutina*) in France. Meanwhile, all ten SSU rRNA gene sequences were identical to each other and showed 97.3% similarity to the *Mermis* sp. reference (FJ973464), previously detected in the *Culicoides obsoletus* midge in the Czech Republic (unpublished data). Therefore, these larvae were identified as mermithid nematodes belonging to the family Mermithidae. The resulting SSU HVR-I and SSU rRNA gene sequences were deposited in GenBank under accession numbers PV490822-PV490831 and PV459227-PV459236, respectively.

**Fig. 7:**
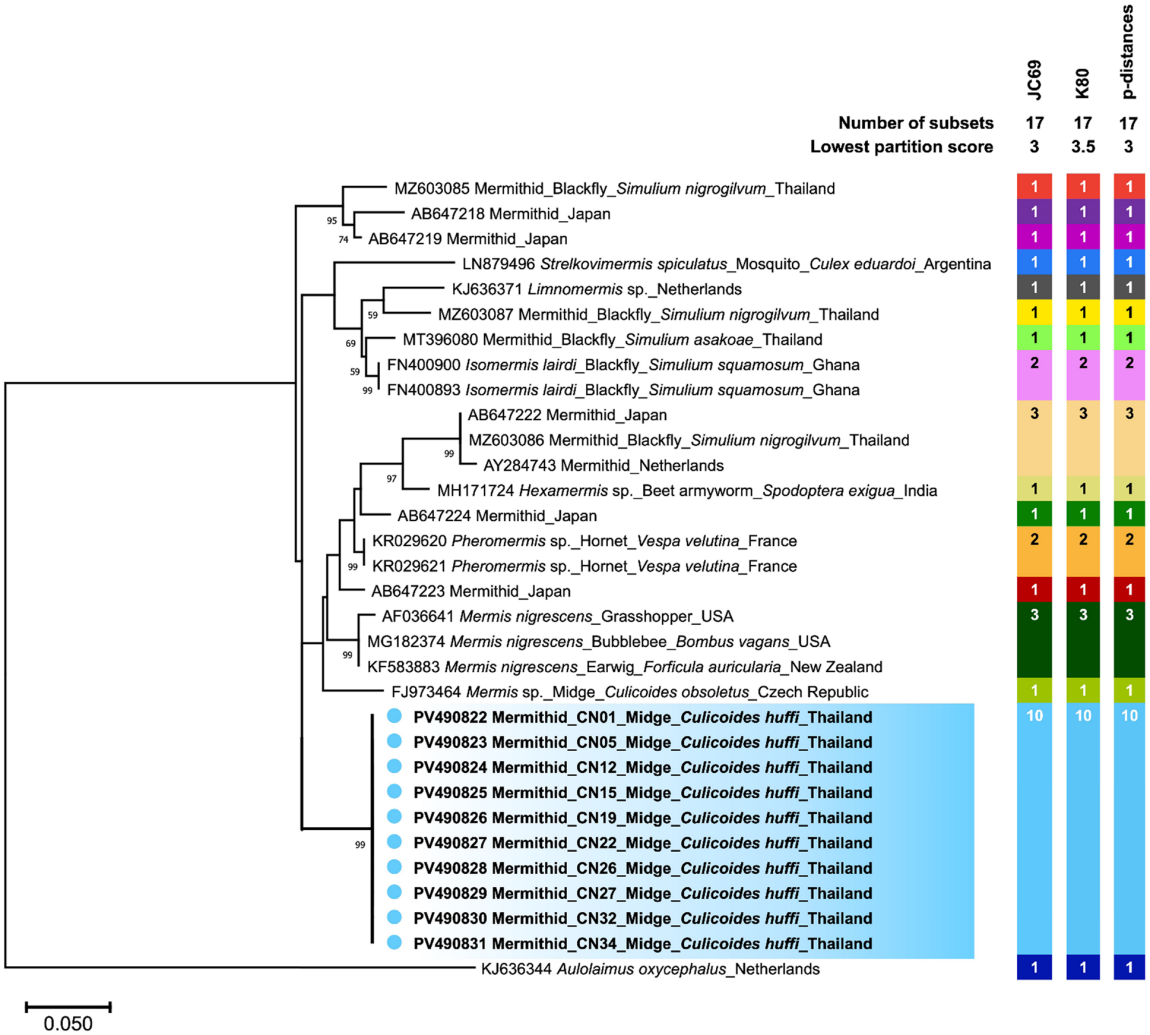
Phylogenetic divergence of mermithid nematodes identified in this study, on the basis of non-filarial SSU HVR-I sequences. The scale bar represents 0.05 nucleotide substitutions per site. ASAP-based species delimitation using three substitution models (JC69, K80, and *p*-distances) with the lowest partition score indicates that our sequences represent a distinct putative species, indicated by blue circles and highlighting. Each colored bar represents the species delimited by the respective substitution model

**Fig. 8:**
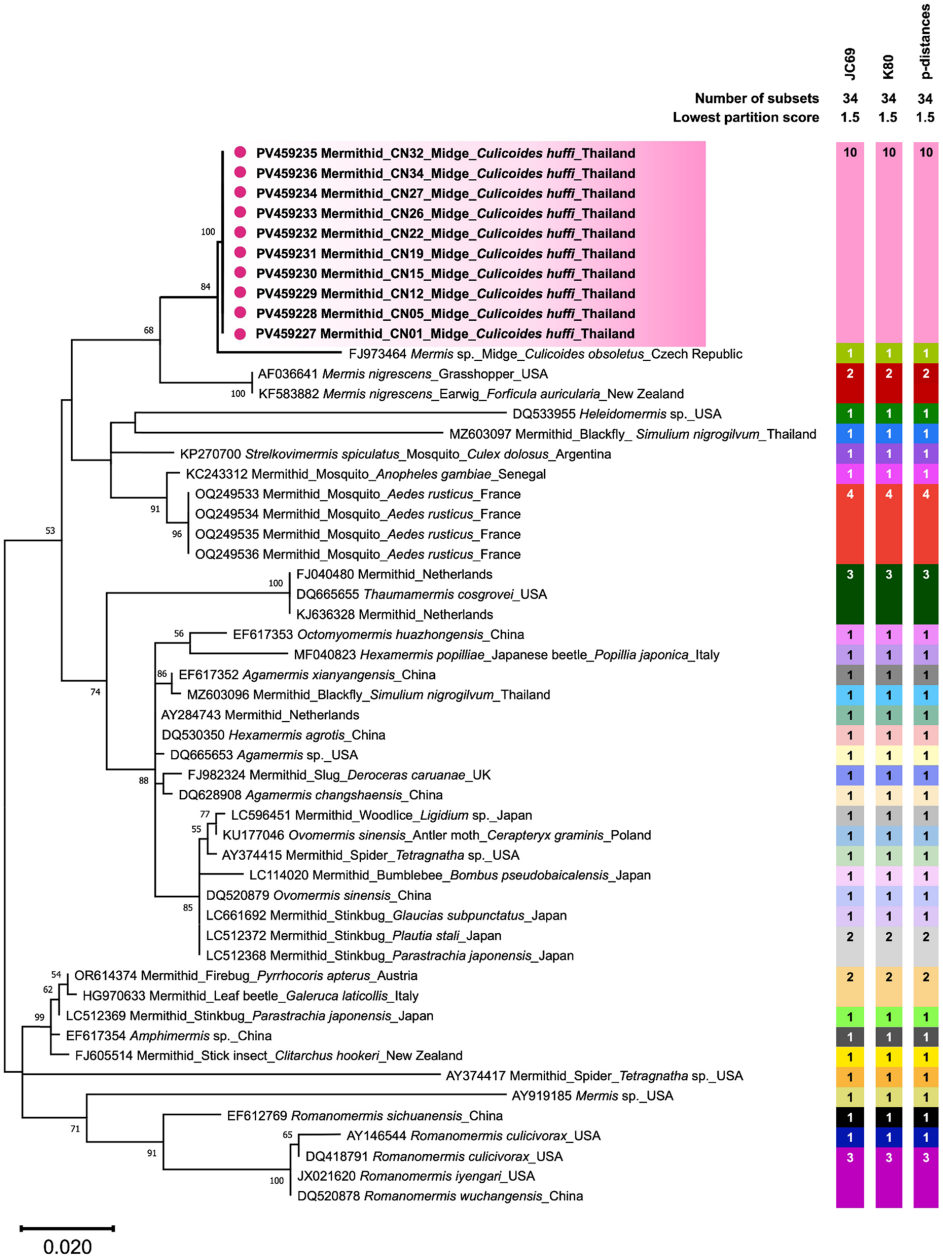
Phylogenetic divergence of mermithid nematodes identified in this study, on the basis of mermithid SSU rRNA gene sequences. The scale bar represents 0.02 nucleotide substitutions per site. Species delimitation using three substitution models with the lowest score assigns our sequences to a single putative species, distinct from others, as indicated by pink circles and highlighting

As shown in Fig. 7, phylogenetic analysis based on SSU HVR-I revealed that our sequences formed a distinct clade separate from all other mermithid species obtained from the GenBank database. Similarly, our SSU rRNA gene sequences were genetically distinct from all other mermithid species included in the phylogenetic analysis (Fig. 8). However, they clustered as a sister group to *Mermis* sp. discovered in *C. obsoletus* (FJ973464) with strong bootstrap support of 84%. This phylogenetic divergence suggests that our mermithid nematodes likely represent a novel species.

Species delimitation of the mermithid SSU HVR-I and SSU rRNA gene sequences was performed using ASAP with three different substitution models on the same sequence dataset used for phylogenetic analysis. Based on the optimal partitioning with the lowest scores among the three models, all sequences from our mermithid samples at both genetic loci were assigned to a single putative species distinct from others, as shown in Figs. 7 and 8. This result is consistent with the phylogenetic analysis described above.

## Discussion

Notably, *Culicoides* biting midges are widely distributed worldwide and serve as important vectors of several pathogens, raising public health concerns for both humans and animals, with significant economic impacts [3, 4]. To address these concerns, controlling vector populations is essential [50–52]. While insecticides provide a fast and convenient method for controlling insect populations, their repeated use can lead to resistance [52, 53]. Additionally, insecticides may cause environmental pollution and pose health risks to animals and humans. Protecting animals using nets, repellents, or air curtains can reduce midge attacks [52, 54]. However, these methods are often not cost-effective and may be impractical for large herds or open fields. Alternatively, biological control offers several advantages, including species specificity, environmental friendliness, safety for animals and humans, and the absence of issues related to chemical resistance [55]. One example is the use of mermithid nematodes, which have been shown experimentally to infect and kill insect larvae, thereby decreasing the reproductive fitness and survival of adult hosts and potentially reducing host populations [21, 22, 30–32]. However, little is known about mermithids infecting *Culicoides* midges, and research on the use of these nematodes for midge biocontrol remains limited.

In the present study, we characterized mermithid parasitism in *Culicoides* specimens collected near the residence of a leishmaniasis patient in Southern Thailand. Interestingly, mermithid infection was found exclusively in adult midges of *C. huffi*, the most abundant species at the time of collection. Ultrastructural examination revealed morphological features characteristic of the juvenile stage, particularly the needle-like stylet and trophosome, which enabled us to identify the mermithids as juveniles [48, 56–58]. The pre-parasitic juvenile (J2) stage is known to penetrate the host using the stylet and develop into the parasitic juvenile stage (J3), which feeds directly on the host’s hemolymph and stores nutrients as the trophosome, as demonstrated by our SEM analysis [59]. Mermithids in the post-parasitic juvenile stage (J4) typically emerge from the host, usually causing host death, before maturing into adults [21, 31, 60].

However, in our study, mermithid juveniles were found in several adult *C. huffi* specimens. This carryover into adult midges may result from late-stage or old midge instar larvae being exposed to pre-parasitic mermithid juveniles [21, 59]. It is also noteworthy that the presence of mermithids in adult hosts has been previously reported in several *Culicoides* species worldwide, including common Southeast Asian species such as *Culicoides jacobsoni, C. obscurus, C. orientalis, C. oxystoma, C. peregrinus,* and *Culicoides shortti* [48]. Prolonged parasitism within adult midges may protect the nematodes from harsh external environmental conditions, including high levels of ammonium and salts [61]. However, infected adult midges may suffer reduced reproductive fitness or increased mortality [62]. They may also exhibit behavioral alterations affecting host-seeking, blood-feeding, mating, and oviposition, which could favor parasite dispersal and transmission, as observed in other parasitized hematophagous insects [21, 35, 63].

Of further interest, most of our mermithid-parasitized midges exhibited morphological abnormalities characteristic of intersex males. These parasitized intersex males were presumably genetically male, possessing typical external male genitalia [21, 23, 33, 34, 64, 65], but their antennae and wing shape resembled those of females. Our results corroborate previous studies reporting the presence of intersexuality associated with mermithid parasitism in several *Culicoides* species, including *Culicoides haematopotus*, *Culicoides crepuscularis*, and *Culicoides stellifer* (USA) [34, 66–69]; *Culicoides cubitalis* (France) [70, 71]; and *Culicoides circumscriptus* (Spain) [72]. It is plausible that the shift in antennal structure and sensilla pattern toward the female form in these parasitized intersex individuals enhances their sensory abilities to locate breeding sites [33]. Additionally, there are significant differences in wing size and shape between the sexes of several *Culicoides* species, suggesting differences in their flight behavior [33, 73]. Unlike males, females require different flight capabilities for dispersal, host-seeking, and locating suitable breeding sites for oviposition and larval development—environments essential for nematodes to mature, reproduce, and infect new midge larvae. Therefore, the feminization of antennae and wing morphology observed in intersex individuals may reflect adaptive structural modifications induced by parasitic nematodes, enabling these midges to fly to breeding sites similarly to gravid females [33]. This adaptation could potentially enhance the reproductive success and transmission of the nematode parasites.

In contrast to the feminized antennae and wing shape, the mouthparts of the intersex males collected in this study exhibited morphological characteristics intermediate between females and males, with the maxillary palps and mandibles more closely resembling those of females. However, this study provides no evidence to confirm that these modifications confer any advantage or are sufficient for functional blood feeding in infected intersex *C. huffi* individuals. Consistent with this, previous studies have found that some mouthpart structures of mermithid-parasitized intersex males in certain *Culicoides* species, such as *C. stellifer* and *C. circumscriptus*, are partially feminized, but no evidence has demonstrated their ability to feed on blood [33, 34, 72]. Importantly, the labrum-epipharynx, hypopharynx, mandibles, and laciniae of the typical female mouthparts function together via an interlocking mechanism to cut the host’s skin, a process essential for blood feeding [74]. Therefore, it can be concluded that the partially modified mouthparts of mermithid-infected intersex males are unlikely to be involved in acquiring energy through blood feeding [33, 34, 72]. Although mermithid parasitism induces partial feminization of mouthpart morphology in several *Culicoides* species, and the nematode might benefit if intersex males could feed on blood, it is more likely that these modifications are simply a consequence of mermithid infection rather than adaptations related to nourishing the parasites through host blood feeding [33].

It has been reported that approximately 40 *Culicoides* species, belonging to 11 subgenera worldwide, are affected by mermithid parasitism [21]. Several mermithid genera infecting *Culicoides* midges at either the immature or adult stage have been recorded, including *Agamomermis*, *Ceratomermis*, *Heleidomermis*, *Mermis*, *Romanomermis*, and *Spiculimermis* [21]. However, the validity of some of these genera remains uncertain and requires further confirmation. Since 1974, mermithids infecting *Culicoides* midges in either the larval or adult stage have generally been classified as belonging to the genus *Heleidomermis* or as undetermined species within the Mermithidae family, solely on the basis of morphological and life history characteristics [21]. It is important to note that mermithid species identification primarily relies on morphological characteristics of the adult stage [21]. Because this study only had access to immature-stage characteristics, morphological identification of the mermithids at the genus or species level was not possible. However, based on our phylogenetic and species delimitation analyses, sequences from two loci likely represent a single putative species distinct from other known mermithid references. Additionally, significant genetic differences were observed when comparing our mermithid sequences with reference sequences, suggesting the presence of a potentially new species. Nonetheless, nucleotide databases such as GenBank and the Barcode of Life Data System (BOLD) contain limited sequence information for mermithids infecting *Culicoides* midges. While sequence analysis indicates the presence of a new species, this conclusion cannot be confirmed due to the lack of adult morphological data and the scarcity of *Culicoides*-infecting mermithid sequences in public databases. Consequently, we have classified our samples as an undetermined species within the Mermithidae family.

Interestingly, *Heleidomermis magnapapula* is known to naturally infect and kill up to 50% of the *C. sonorensis* larvae in dairy wastewater ponds in Southern California [21]. In a previous experimental release trial, the number of adult *C. sonorensis* emerging from field enclosures decreased by 84% after 40,000 mermithid pre-parasitic juveniles of *H. magnapapula* were introduced into each enclosure containing 1500 laboratory-reared *C. sonorensis* eggs [21]. This substantial reduction in emerging midges is most likely due to premature death caused by the invasion of numerous infective pre-parasitic juveniles into the midge larvae. Furthermore, the application of mermithids for vector control has been more extensively studied in mosquitoes. Experiments using the mermithid *Romanomermis* have demonstrated significant reductions in mosquito populations, with infection rates of up to 60% [31, 32, 35, 36, 75]. Although these experimental results suggest the potential of mermithids for controlling *Culicoides* midges, evidence of effective biocontrol in natural midge populations remains lacking. Several challenges also limit the use of mermithids for insect control. First, stable laboratory colonization of local *Culicoides* species is essential to enable host-based mass rearing of mermithids, which is necessary to produce millions of infective juveniles weekly [21, 76, 77]. Such large-scale production is required to adequately cover extensive midge breeding areas. However, locating the natural breeding sites of both *Culicoides* and mermithids in the wild is very difficult, making the use of these parasites for control impractical. Additionally, the complex population dynamics of *Culicoides*, which vary geographically and temporally, combined with the high host specificity of mermithids, may further restrict the feasibility of this biocontrol approach [15, 21, 78].

To our knowledge, this is the first report of mermithid parasitism in adult *Culicoides* midges in Thailand. These novel morphological and molecular findings will enhance our understanding of the biology of mermithid nematodes that parasitize *Culicoides* midges. However, further research is needed to investigate the diversity and life cycle of mermithids within the *Culicoides* community, as well as their impacts on hosts. Such studies will be valuable for evaluating the potential of these entomopathogenic nematodes as biological agents against midge populations.

## Conclusions

This study provides the first evidence of mermithid parasitism in *Culicoides* midges in Thailand, utilizing both ultrastructural and molecular analyses. The new morphological and molecular findings presented here enhance our understanding of the biology of these entomopathogenic nematodes, which remain underexplored. Given the current lack of evidence supporting mermithid-based biocontrol of midges in natural settings, further research is needed to assess the potential of these nematodes as part of integrated vector-control strategies aimed at reducing midge populations.

## Supplementary Information

Supplementary material 1. Table S1. SSU HVR-I sequences of mermithid nematodes included for phylogenetic analysis.

Supplementary material 2. Table S2. SSU rRNA gene sequences of mermithid nematodes included for phylogenetic analysis.

## Data Availability

All data supporting the results of this study are available in the paper and its supplementary information. The mermithid SSU HVR-I and SSU rRNA gene sequences obtained from this study have been deposited into the GenBank database under accession numbers PV490822-PV490831 and PV459227-PV459236, respectively.

## Abbreviations

dsDNA: Double-stranded DNA
MEGA: Molecular evolutionary genetics analysis
MU UV-LED: Mahidol University ultraviolet light-emitting diodes
PCR: Polymerase chain reaction
