# Supplementary material for: First record of mermithid parasitism in adult biting midges, Culicoides huffi (Diptera: Ceratopogonidae), collected from Southern Thailand, with ultrastructural and molecular characterization
Source: Parasit Vectors. 2025 Jul 28;18:303. doi: 10.1186/s13071-025-06958-x (PMC12302758; doi:10.1186/s13071-025-06958-x)
Supplement: Supplementary file 1 — Supplementary material 1. Table S1. SSU HVR-I sequences of mermithid nematodes included for phylogenetic analysis. [file 13071_2025_6958_MOESM1_ESM.docx]

**Supplementary File 1: Table S1**. SSU HVR-I sequences of mermithid nematodes included for phylogenetic analysis

| **Accession no.** | **Species** | **Host** | **Country** | **Reference** |
| --- | --- | --- | --- | --- |
| MH171724 | *Hexamermis* sp. | Beet armyworm  (*Spodoptera exigua*) | India | [1] |
| FN400900 | *Isomermis lairdi* | Blackfly  (*Simulium squamosum*) | Ghana | [2] |
| FN400893 | *Isomermis lairdi* | Blackfly  (*Simulium squamosum*) | Ghana | [2] |
| KJ636371 | *Limnomermis* sp. | - | Netherlands | [3] |
| AF036641 | *Mermis nigrescens* | Grasshopper | USA | [4] |
| MG182374 | *Mermis nigrescens* | Bubble bee  (*Bombus vagans*) | USA | [5] |
| KF583883 | *Mermis nigrescens* | Earwig  (*Forficula auricularia*) | New Zealand | [6] |
| FJ973464 | *Mermis* sp. | Midge  (*Culicoides obsoletus*) | Czech Republic | Unpublished |
| AY284743 | Undetermined species of the Mermithidae family | - | Netherlands | [3] |
| AB647218 | Undetermined species of the Mermithidae family | - | Japan | [7] |
| AB647219 | Undetermined species of the Mermithidae family | - | Japan | [7] |
| AB647222 | Undetermined species of the Mermithidae family | - | Japan | [7] |
| AB647223 | Undetermined species of the Mermithidae family | - | Japan | [7] |
| AB647224 | Undetermined species of the Mermithidae family | - | Japan | [7] |
| MT396080 | Undetermined species of the Mermithidae family | Blackfly  (*Simulium asakoae*) | Thailand | [8] |
| MZ603085 | Undetermined species of the Mermithidae family | Blackfly  (*Simulium nigrogilvum*) | Thailand | [9] |
| MZ603086 | Undetermined species of the Mermithidae family | Blackfly  (*Simulium nigrogilvum*) | Thailand | [9] |
| MZ603087 | Undetermined species of the Mermithidae family | Blackfly  (*Simulium nigrogilvum*) | Thailand | [9] |
| KR029620 | *Pheromermis* sp. | Hornet (*Vespa velutina*) | France | [10] |
| KR029621 | *Pheromermis* sp. | Hornet (*Vespa velutina*) | France | [10] |
| LN879496 | *Strelkovimermis spiculatus* | Mosquito (*Culex eduardoi*) | Argentina | [11] |
| KJ636344 | *Aulolaimus oxycephalus*  (outgroup) | - | Netherlands | [3] |

**References (Table S1)**

1. Babu SR, Phani V, Meena PK, Chauhan K, Khan MR, Somvanshi VS. Report of a mermithid nematode infecting *Amyna axis*, *Chrysodeixis* spp. and *Spodoptera* spp. from India. Journal of Biol Control. 2019;33:217-221.
2. Crainey JL, Wilson MD, Post RJ. An 18S ribosomal DNA barcode for the study of *Isomermis lairdi*, a parasite of the blackfly *Simulium damnosum s.l.* Med Vet Entomol. 2009;23:238-244.
3. Holterman M, van der Wurff A, van den Elsen S, van Megen H, Bongers T, Holovachov O, et al. Phylum-wide analysis of SSU rDNA reveals deep phylogenetic relationships among nematodes and accelerated evolution toward crown Clades. Mol Biol Evol. 2006;23:1792-1800.
4. Blaxter ML, De Ley P, Garey JR, Liu LX, Scheldeman P, Vierstraete A, et al. A molecular evolutionary framework for the phylum Nematoda. Nature. 1998;392:71-75.
5. Tripodi AD, Strange JP. Rarely reported, widely distributed, and unexpectedly diverse: molecular characterization of mermithid nematodes (Nematoda: Mermithidae) infecting bumble bees (Hymenoptera: Apidae: Bombus) in the USA. Parasitology. 2018;145:1558-1563.
6. Presswell B, Evans S, Poulin R, Jorge F. Morphological and molecular characterization of *Mermis nigrescens* Dujardin, (Nematoda: Mermithidae) parasitizing the introduced European earwig (Dermaptera: Forficulidae) in New Zealand. J Helminthol. 2015;89:267-276.
7. Sato T, Watanabe K, Tamotsu S, Ichikawa A, Schmidt-Rhaesa A. Diversity of nematomorph and cohabiting nematode parasites in riparian ecosystems around the Kii Peninsula, Japan. Can J Zool. 2012;90:868-878.
8. Aupalee K, Saeung A, Srisuka W, Fukuda M, Streit A, Takaoka H. Seasonal filarial infections and their black fly vectors in Chiang Mai Province, Northern Thailand. Pathogens. 2020;9:512.
9. Huang F, Srisuka W, Aupalee K, Streit A, Fukuda M, Pitasawat B, et al. Diversity of nematodes infecting the human-biting black fly species, *Simulium nigrogilvum* (Diptera: Simuliidae) in central Thailand. Acta Trop. 2021;224:106140.
10. Villemant C, Zuccon D, Rome Q, Muller F, Poinar GO Jr, Justine JL. Can parasites halt the invader? Mermithid nematodes parasitizing the yellow-legged Asian hornet in France. PeerJ. 2015;3:e947.
11. Lopez RP, Díaz-Nieto LM, Berón CM. New host and distribution for the mosquito parasite *Strelkovimermis spiculatus*. Rev Soc Entomol Argent. 2016;75:101-104.
