# Supplementary material for: First record of mermithid parasitism in adult biting midges, Culicoides huffi (Diptera: Ceratopogonidae), collected from Southern Thailand, with ultrastructural and molecular characterization
Source: Parasit Vectors. 2025 Jul 28;18:303. doi: 10.1186/s13071-025-06958-x (PMC12302758; doi:10.1186/s13071-025-06958-x)
Supplement: Supplementary file 2 — Supplementary material 2. Table S2. SSU rRNA gene sequences of mermithid nematodes included for phylogenetic analysis. [file 13071_2025_6958_MOESM2_ESM.docx]

**Supplementary File 2: Table S2**. SSU rRNA gene sequences of mermithid nematodes included for phylogenetic analysis

| **Accession no.** | **Species** | **Host** | **Country** | **Reference** |
| --- | --- | --- | --- | --- |
| DQ628908 | *Agamermis changshaensis* | - | China | Unpublished |
| DQ665653 | *Agamermis* sp. | - | USA | Unpublished |
| EF617352 | *Agamermis xianyangensis* | - | China | Unpublished |
| EF617354 | *Amphimermis* sp. | - | China | Unpublished |
| DQ533955 | *Heleidomermis* sp. | - | USA | Unpublished |
| DQ530350 | *Hexamermis agrotis* | - | China | Unpublished |
| MF040823 | *Hexamermis popilliae* | Japanese beetle  (*Popillia japonica*) | Italy | [1] |
| AF036641 | *Mermis nigrescens* | Grasshopper | USA | [2] |
| KF583882 | *Mermis nigrescens* | Earwig  (*Forficula auricularia*) | New Zealand | [3] |
| FJ973464 | *Mermis* sp. | Midge  (*Culicoides obsoletus*) | Czech Republic | Unpublished |
| AY919185 | *Mermis* sp. | - | USA | [4] |
| AY284743 | Undetermined species of the Mermithidae family | - | Netherlands | [5] |
| FJ040480 | Undetermined species of the Mermithidae family | - | Netherlands | [5] |
| KJ636328 | Undetermined species of the Mermithidae family | - | Netherlands | [5] |
| MZ603096 | Undetermined species of the Mermithidae family | Blackfly  (*Simulium nigrogilvum*) | Thailand | [6] |
| MZ603097 | Undetermined species of the Mermithidae family | Blackfly  (*Simulium nigrogilvum*) | Thailand | [6] |
| LC114020 | Undetermined species of the Mermithidae family | Bubble bee  (*Bombus pseudobaicalensis*) | Japan | [7] |
| HG970633 | Undetermined species of the Mermithidae family | Leaf beetle  (*Galeruca laticollis*) | Italy | [8] |
| OQ249533 | Undetermined species of the Mermithidae family | Mosquito (*Aedes rusticus*) | France | [9] |
| OQ249534 | Undetermined species of the Mermithidae family | Mosquito (*Aedes rusticus*) | France | [9] |
| OQ249535 | Undetermined species of the Mermithidae family | Mosquito (*Aedes rusticus*) | France | [9] |
| OQ249536 | Undetermined species of the Mermithidae family | Mosquito (*Aedes rusticus*) | France | [9] |
| KC243312 | Undetermined species of the Mermithidae family | Mosquito  (*Anopheles gambiae*) | Senegal | [10] |
| FJ982324 | Undetermined species of the Mermithidae family | Slug  (*Deroceras caruanae*) | UK | [11] |
| AY374415 | Undetermined species of the Mermithidae family | Spider (*Tetragnatha* sp.) | USA | [12] |
| AY374417 | Undetermined species of the Mermithidae family | Spider (*Tetragnatha* sp.) | USA | [12] |
| FJ605514 | Undetermined species of the Mermithidae family | Stick insect  (*Clitarchus hookeri*) | New Zealand | [13] |
| LC661692 | Undetermined species of the Mermithidae family | Stinkbug  (*Glaucias subpunctatus*) | Japan | [14] |
| LC512368 | Undetermined species of the Mermithidae family | Stinkbug  (*Parastrachia japonensis*) | Japan | [15] |
| LC512369 | Undetermined species of the Mermithidae family | Stinkbug  (*Parastrachia japonensis*) | Japan | [15] |
| LC512372 | Undetermined species of the Mermithidae family | Stinkbug (*Plautia stali*) | Japan | [15] |
| OR614374 | Undetermined species of the Mermithidae family | Firebug  (*Pyrrhocoris apterus*) | Austria | [16] |
| LC596451 | Undetermined species of the Mermithidae family | Woodlice (*Ligidium* sp.) | Japan | [17] |
| EF617353 | *Octomyomermis*  *huazhongensis* | - | China | Unpublished |
| DQ520879 | *Ovomermis sinensis* | - | China | Unpublished |
| KU177046 | *Ovomermis sinensis* | Antler moth  (*Cerapteryx graminis*) | Poland | Unpublished |
| DQ418791 | *Romanomermis culicivorax* | - | USA | Unpublished |
| AY146544 | *Romanomermis culicivorax* | - | USA | [18] |
| JX021620 | *Romanomermis iyengari* | - | USA | Unpublished |
| EF612769 | *Romanomermis sichuanensis* | - | China | Unpublished |
| DQ520878 | *Romanomermis wuchangensis* | - | China | Unpublished |
| KP270700 | *Strelkovimermis spiculatus* | Mosquito (*Culex dolosus*) | Argentina | [19] |
| DQ665655 | *Thaumamermis cosgrovei* | - | USA | Unpublished |

**References (Table S2)**

1. Mazza G, Paoli F, Strangi A, Torrini G, Marianelli L, Peverieri GS, et al. *Hexamermis popilliae* n. sp. (Nematoda: Mermithidae) parasitizing the Japanese beetle *Popillia japonica* Newman (Coleoptera: Scarabaeidae) in Italy. Syst Parasitol. 2017;94:915-926.
2. Blaxter ML, De Ley P, Garey JR, Liu LX, Scheldeman P, Vierstraete A, et al. A molecular evolutionary framework for the phylum Nematoda. Nature. 1998;392:71-75.
3. Presswell B, Evans S, Poulin R, Jorge F. Morphological and molecular characterization of *Mermis nigrescens* Dujardin, (Nematoda: Mermithidae) parasitizing the introduced European earwig (Dermaptera: Forficulidae) in New Zealand. J Helminthol. 2015;89:267-276.
4. Powers TO, Todd TC, Burnell AM, Murray PCB, Fleming CC, Szalanski AL, et al. Incorporating molecular identification of *Meloidogyne* spp. into a large-scale regional nematode survey. J Nematol. 2005;37:226-235.
5. Holterman M, van der Wurff A, van den Elsen S, van Megen H, Bongers T, Holovachov O, et al. Phylum-wide analysis of SSU rDNA reveals deep phylogenetic relationships among nematodes and accelerated evolution toward crown Clades. Mol Biol Evol. 2006;23:1792-1800.
6. Huang F, Srisuka W, Aupalee K, Streit A, Fukuda M, Pitasawat B, et al. Diversity of nematodes infecting the human-biting black fly species, *Simulium nigrogilvum* (Diptera: Simuliidae) in central Thailand. Acta Trop. 2021;224:106140.
7. Kubo R, Ugajin A, Ōno M. Molecular phylogenetic analysis of mermithid nematodes (Mermithida: Mermithidae) discovered from Japanese bumblebee (Hymenoptera: Bombinae) and behavioral observation of an infected bumblebee. Appl Entomol Zool. 2016;51:549-554.
8. Poinar GO Jr, Welch HA. Nematode isolated from *Galeruca laticollis* (Coleoptera: Chrysomelidae). J Invertebr Pathol. 1968;12:280-283.
9. Martinet JP, Aatif I, Depaquit J. Three *Aedes* species infested by mermithids in France. Parasite. 2023;30:12.
10. Kobylinski KC, Sylla M, Black W, Foy BD. Mermithid nematodes found in adult *Anopheles* from southeastern Senegal. Parasit Vectors. 2012;5:131.
11. Ross JL, Ivanova ES, Spiridonov SE, Waeyenberge L, Moens M, Nicol GW, et al. Molecular phylogeny of slug-parasitic nematodes inferred from 18S rRNA gene sequences. Mol Phylogenet Evol. 2010;55:738-743.
12. Vandergast AG, Roderick GK. Mermithid parasitism of Hawaiian *Tetragnatha* spiders in a fragmented landscape. J Invertebr Pathol. 2003;84:128-137.
13. Yeates GW, Buckley TR. First records of mermithid nematodes (Nematoda: Mermithidae) parasitising stick insects (Insecta: Phasmatodea). New Zeal J Zool. 2010;37:279-283.
14. Watanabe S, Tsunashima A, Itoyama K, Shinya R. Survey of mermithid nematodes (Mermithida: Mermithidae) infecting fruit-piercing stink bugs (Hemiptera: Pentatomidae) in Japan. Appl Entomol Zool. 2021;56:27-39.
15. Iryu, T., Tanaka, R., & Yoshiga, T. Mermithid nematodes isolated from the shield bug Parastrachia japonensis. Nematol Res. 2020;50:1-7.
16. Kostygov AY, Chmelová Ľ, Reichl J, Jászayová A, Votýpka J, Fuehrer HP, et al. Parasites of firebugs in Austria with focus on the "micro"-diversity of the cosmopolitan trypanosomatid *Leptomonas pyrrhocoris*. Parasitol Res. 2023;123:27.
17. Yoshino H, Waki T. First report on Mermithidae (Mermithida) infection in *Ligidium* sp. (Isopoda, Ligiidae). Parasitol Int. 2021;82:102304.
18. Mullin PG, Harris TS, Powers TO. Phylogenetic relationships of *Nygolaimina* and *Dorylaimina* (Nematoda: Dorylaimida) inferred from small subunit ribosomal DNA sequences. Nematology. 2005;7:59-79.
19. Belaich MN, Buldain D, Ghiringhelli PD, Hyman B, Micieli MV, Achinelly MF. Nucleotide sequence differentiation of Argentine isolates of the mosquito parasitic nematode *Strelkovimermis spiculatus* (Nematoda: Mermithidae). J Vector Ecol. 2015;40:415-418.
